# “…*He’s not beating me*”—Socio-cultural construction of intimate partner violence and traditional birth attendants: implications for maternal & child health in rural communities in Hohoe, Ghana

**DOI:** 10.3389/fgwh.2024.1352793

**Published:** 2024-03-19

**Authors:** Titilayo A. Okoror, Bless M. Nyamuame, Cordelia Martin-Ikpe, Yasmeen Gilani, Samuel Elikem Nyamuame

**Affiliations:** ^1^ Department of Africana Studies & Global Public Health Program, Binghamton, NY, United States; ^2^ Center for Health Equity and Evaluation Research (CHEER), School of Public Health, Texas A&M University, College Station, TX, United States; ^3^ Department of Midwifery, Nursing & Midwifery Training College, Hohoe, Ghana; ^4^ Department of Africana Studies, Binghamton University (SUNY), Vestal Parkway East, Binghamton, NY, United States

**Keywords:** intimate partner violence, nursing mothers, traditional birth attendants, maternal and child health, socio-cultural construction, Ghana

## Abstract

**Introduction:**

Most research on Intimate Partner Violence (IPV) focuses on the physical, sexual and psychological abuse, with less focus on the financial abuse. This study explores nursing mothers' experiences and perceptions of financial and material support from their significant others and traditional birth attendants' (TBA) observations of support to nursing mothers in their communities.

**Methods:**

Using purposive sampling, focus groups and interviews were conducted primarily in Ewe language among nursing mothers and TBAs in rural communities in Hohoe, Volta region, Ghana. All discussions were audio-recorded and transcribed for analysis. Thematic analysis guided by the social constructivist framework was used in data analysis.

**Results:**

Twenty-seven women participated in the study, ranging in ages from 19 to 82 (20 nursing mothers; 7 TBAs). Most participants were married (19) and about 65% reported working outside the home (10 nursing mothers; 7 TBAs). Two themes emerged from the data analysis: Lack of support from partners for housekeeping chores and finances; and TBAs as mediators. Nursing mothers who reported lack of financial support did not perceive it as abuse, rather as hinderance to their efforts to care for their children. TBAs act as mediators interceding on behalf of nursing mothers with their husbands and fathers of their children, while also seeking resources to support them.

**Discussion:**

Understanding the perceptions and socio-cultural meanings women attached to IPV experience is essential for effective intervention to reduce IPV. In addition, TBAs can be a resource in intervening to alleviate IPV in their communities, thereby improving maternal and child health.

## 1 Introduction

Intimate Partner Violence (IPV) is described as a behavior by a current or former intimate partner that “causes physical, sexual or psychological harm, including acts of physical aggression, sexual coercion, psychological abuse and controlling behaviors” (1). Physical violence can involve hitting, kicking, burning, punching, shoving, slapping, hair-pulling, biting or forcing of alcohol and/or drug, or other physical force. Sexual violence is any sexual act committed against the will of another person or when the person does not give consent or when consent cannot be given due to mental disability, severe intoxication or unconsciousness due to drug or alcohol or the person is a child. Emotional abuse can entail erosion of the person's self-esteem due to constant belittling, criticism, verbal abuse, and isolation from friends and family (2). It is estimated that 736 million women globally have experienced physical and/or sexual intimate partner violence, non-partner sexual violence, or both in their life, while more than 640 million women aged 15 and older have been subjected to intimate partner violence (2). The World Bank estimated that 30% of women have experienced intimate partner violence or non-partner sexual violence, with Sub-Saharan Africa having one of the highest prevalence of IPV where 33% of women aged 15–49 years suffered IPV in their lifetime (3). Despite the disparities across countries in Sub-Saharan Africa, research on prevalence and associated factors of IPV reported physical violence at 30.58%, emotional violence at 30.22% and sexual violence at 42.62% (4). In Ghana, a west African country, reports indicate that 24.4% of women aged 15–49 years have experienced lifetime physical and/or sexual intimate partner violence, while 19.2% of the same age group have experienced physical and/or sexual intimate partner violence in the last 12 months (2). Intimate Partner Violence effects on women and girls have far-reaching long-term implications (5–11), and can be more devastating when experienced during pregnancy (12–18). Women who experienced IPV during pregnancy had a lower rate of receiving adequate antenatal care and skilled delivery, and were more likely never to enter care and/or delay care (19, 20). And in rural communities where skilled delivery are non-existent, traditional birth attendants are usually the only resource available to pregnant women.

Traditional Birth Attendants (TBAs) are described as individuals who have acquired their skills by delivering babies themselves or through apprenticeship to other TBAs, and now assist mothers during childbirth (21). In most cases, TBAs do not have biomedical training, rather they are self-taught through personal experience, spiritual gifting or training by another TBA (22). Most TBAs assist in births as a secondary occupation, which is also how many acquire their skills and knowledge (22, 23). Kennedy (24) describes most TBAs as specialists only in obstetrics, though they may provide basic health care and support, and share basic information they acquired through traditions and practices of the communities they serve (25). Since they are usually members of the community they serve, they are readily accessible and available day and night to community members (26). In the 1980s, TBAs availability and accessibility to community members became one of the factors for encouraging their integration into the healthcare system in limited resource settings especially in rural communities where skilled birth attendants (nurses, midwives, doctors) are unavailable. Trainings were provided to TBAs on effective hygiene practices such as handwashing, use of clean scissors, and ways to recognize signs of obstetric complications for quick referral to skilled birth attendants (22, 26). However, by the 1990s, reports by international organizations on maternal and child health led to the end of training programs for TBAs (21, 27). Ghana, like other developing countries, decided to ban TBAs services, despite the fact that they are usually the first point of care access for pregnant women in rural communities. Ghana government abandoned their nation-wide reorientation program for TBAs and discourage their use entirely, although the Danfa rural health program evaluation that included the training of TBAs demonstrated that it had a positive impact on maternal health (22, 28). In addition, TBAs provide auxiliary services such as psychological and emotional support that go beyond care and delivery to improve maternal and child health. While there is consensus in research as to the impact of physical, sexual and emotional abuse on maternal and child health, and the role of low socioeconomic status as a precursor to physical, emotional and sexual abuse (29–31), the socio-cultural meaning attached to such experiences are yet to be explored. Specifically, how do women perceive “abuse” if it is not physical, emotional or sexual in nature?

The overarching goal of this study was to explore the role of traditional birth attendants (TBA) in maternal and child health in Hohoe, Ghana, despite the government policy banning their services. Research indicated that pregnant women, especially in rural communities still use TBA for care and delivery despite the ban, and TBAs are forced to practice away from the purview of the government (26, 32–35). While there is research on the impact of the ban on TBAs and women who sought their care in the western, central, and Greater Accra region of Ghana (26, 34, 36), there is dearth of research in the Volta region of Ghana. In addition, this study explored challenges experienced by nursing mothers, in terms of care and support from their partners, family, community and healthcare institutions. Also, it explored traditional birth attendants' observations of IPV of nursing mothers in their communities. The study was approved by the Binghamton University institutional review board.

Hohoe municipal district is one of the 25 administrative districts of the Volta Region of Ghana. According to the 2010 census released in 2014, the municipality had a population of 167,000 with females making up over 52% (37). Over 89% of the population identifies as Christians, with majority of the population engaging in petty trade, crop farming and livestock keeping. It is estimated that 65.3% of rural localities are agricultural households (six out of every ten households) (37). While 43% of the population are married, almost 6% are in informal/consensual union/living together. Fourteen percent of married population have no education, almost 63% have basic education, 10.2% have secondary education and 13% have post-secondary higher levels of education (37).

## 2 Methods & materials

In collaboration with local nurses with ties to rural communities, a workshop entitled, “*Healthy Mothers, Healthy Babies*” was organized to encourage nursing mothers to visit the Child Welfare Centers (CWC) closest to their communities. The workshop had three lesson modules: family planning, immunization, and home management of diarrhea. The program was advertised through personal networks of local nurses to nursing mothers in rural areas close to the Child Welfare Center in the community. The goal was to ensure that the location was convenient, known and accessible to most women in the community. The workshop was presented by local nurses mostly in the Ewe language and some English. Following the workshop, announcement about the study, its purpose and location was made by the lead author. Interested participants were told to contact one of the research assistants on the project through the phone number provided. Traditional birth attendants were contacted through personal networks of the local nurses and some of the nursing mothers that attended the workshop. Traditional birth attendants were provided with information about the study, along with telephone number to call if they were interested. Interviews and focus group discussions with nursing mothers took place three days after the workshop while interviews and focus group discussions with traditional birth attendants took place a week after the workshop. All data collection activities took place at a school classroom in the community, convenient to participants.

Nursing mothers and traditional birth attendants that contacted the research assistants were provided with the location and time. Before the beginning of the discussions and interviews, the second author read the inform consent in Ewe language, and each participant was assisted by the research assistants to sign the consent form and complete the demographic survey. The demographic survey had questions about age, marital status, religious group, local district/region, number of children, employment status and household socioeconomic status. Discussions and interviews with nursing mother were guided by three questions that explored their experience of seeking care during pregnancy, their experience of child birth, and the care and support from their partner. Interviews and discussions with TBAs were also guided by three questions that explored their experience since the ban went into effect, challenges they observe among women (pregnant/nursing mothers) seeking care, and their recommendations on how to improve maternal and child care. Discussion questions were translated into Ewe, back-translated into English and then Ewe to ensure main ideas are conveyed. Three focus groups and two interviews were conducted: two focus groups discussions with nursing mothers, and one focus group discussions and two interviews with traditional birth attendants. Participants were compensated for their time. Discussions and interviews were conducted in Ewe language and some English, and were audio-recorded. The focus groups discussions lasted for 1.5 h, while the interviews lasted for 45 min. The research team also took extensive fieldnotes.

## 3 Data analysis

All audio-recordings were transcribed into Ewe then translated into English. Transcribing and translation of the data was carried out by an independent consultant. Translation was verified by the fifth author, confirmed by the second and first authors, and augmented with research team's field notes. A hybrid approach of inductive and deductive method was employed during coding (38). Deductive approach was used in coding participants' responses to discussion questions, while inductive approach guided themes that emerged from responses, examples and explanations provided by participants to expatiate on ideas (38). Thematic analysis guided by the social constructivist framework was used in the data analysis. The social constructivism framework posits that individuals develop subjective meanings of their experiences through interactions with others, their historical and cultural contexts (39). These meanings are not innate in individuals, rather they are negotiated socially as individuals engage with others and the world around them, and develop meanings to correspond to their reality (39).

In line with the suggested three phases and seven stages of thematic analysis by Swain (38), the first and second authors engaged in a complete immersion in the data through constant reading and re-reading, generated initial codes using deductive and inductive methods, which were then compared for inter-coder reliability. Codes were coalesced to create themes that were constantly reviewed to ensure interpretation of meaning, while using quotes from participants' responses to define identified themes. It must be stated that the approach was iterative, and some stages occur concurrently (38, 40). Use of the social constructivism allowed for interpretation of participants' responses within their socio-cultural contexts and worldview. In addition, the research team, specifically the second and first authors were actively involved with participants in making sense of the meanings of their experiences of childbirth, perceptions of partners' support or lack therefore, and their roles as traditional birth attendants in their communities despite the ban. Also, having a shared cultural experience and language with participants allowed for reflexivity for the researchers.

## 4 Results

Twenty-seven women participated in the study—20 nursing mothers and seven traditional birth attendants. Nursing mothers’ age ranges from 18 to 40 years old, while traditional birth attendants' age ranges from 25 to 82 (see Table 1). Most participants were married (19) and about 65% reported working outside the home (10 nursing mothers; 7 TBAs). Among nursing mothers, 50% (5) of those who reported working also reported not having enough money for basic things like food and clothes, while all TBAs reported having money for food and clothes but short on many other things. On the other hand, only three participants among nursing mothers who reported not working also reported not having enough money for basic things like food and clothes. In addition, 9 out of the 16 married nursing mothers reported having enough money for foods and clothes, but short on many other things. Only three nursing mothers reported having more than secondary school education, while 10 reported primary education or less. Among traditional birth attendants, only three reported primary education or less, and years of practice as TBA ranges from 1 to 60 years. All the participants were mothers with children. Two main themes emerged from the data analysis: (1) Lack of support from partners for housekeeping chores and finances; and (2) Traditional birth attendants as Mediators.

**Table 1:** Socio-demographic characteristics of participants.

Characteristic	Nursing mothers (*N* = 20)	Traditional birth attendants (*N* = 7)
Age range	18–40 years	25–82 years
Marital status		
Married	16	3
Not married	4	7
Working outside home		
Yes	10	7
No	10	
Education		
None	7	4
Primary sch or less	10	3
Post-secondary	3	
Household situation		
Not enough money for basic things like food and clothes	8	
Have money for food and clothes but short on many other things	12	7

### 4.1 Lack of support from partners for housekeeping chores and finances

Participants discussed the lack of support in caring for housekeeping chores by their partners especially during their pregnancy. Many reported having to engage in strenuous house work though pregnant, and in many instances returning to the same situation shortly after giving birth as explained below:

“When I was pregnant, from the first month, I started vomiting, I could not take in anything… Moreover, with all these difficulties, I work as a porter or an aid at a local restaurant so I continued to go to work although the food been prepared are always nauseating. As a result, I vomit throughout the period or the day. But later, the vomiting ceased and I resorted to doing everything for myself. Although, my husband was there, he hasn't helped.” [Nursing Mother #4]

“I have a good relationship with the nurses during the antenatal period. They gave me all the care I needed. I have been cooking by myself and do all activities by myself. My husband was not helping.” [Nursing Mother #3]

“He does not help with the house work… I do everything by myself. I cook, bath the new born baby and every other thing. I do everything by myself though I was pregnant” [Nursing Mother #20]

“…my husband was with me…For cooking, no matter what he will not be ready to do it. Anytime I am having problem with the pregnancy, I have to go the hospital myself until when he realizes that the situation is becoming serious before he will do the follow up to the hospital to find out what is happening.” [Nursing Mother #15]

“I am 18 years old. I did not experience any difficulty during my pregnancy period. I was salivating and that caused me to spit around a lot. They have given me some malaria medications at the hospital but any time I take it, I feel very weak…[Whom were you living with during this period?] I live with my husband. [What kind of support does he give you?]…He does not do anything for me. I do virtually everything. I cook, wash and do everything. [So, at home, do you live with only your husband or were there other people?] There are his siblings also living in the house but they don't offer any assistance.” [Nursing Mother #7]

As indicated by some participants, family members can, in some cases contribute negatively to women's experience of pregnancy and childbirth. A participant experience of the dissolution of her relationship due to the role of her mother-in-law exemplified this as explained below:

“When I became pregnant, my husband said I should abort it. But I decided to keep it.”

Q: Is it the first baby?

RES: No. The second baby

Q:…Is it the spacing of the children?

RES: No. There is 5 years space between the first and second child. He said that his mother said I should not give birth to any other child for him…

Q: Has your husband gotten married to another woman?

RES: I am the only woman with him. His mother said he should not marry someone from the Ewe tribe (Anlo) but from her tribe. So, he left the house for his mother's place.

Q: Is he aware that you gave birth?

RES: He is aware that I have delivered. Someone called to inform him that I have delivered…he has not come back and does not ask of the baby

Q: What about your family?

RES: My father is not here [in the community], and my mother is disabled and cannot walk. The rest of [my] family said they did not ask me to get married. [Nursing Mother #11]

Comments from traditional birth attendants confirmed nursing mothers experience of lack of support from their partners as described below:

“Some [husbands] do not help their wives so I give advice to those men who do not support their wives. I tell them what they are to do during pregnancy and delivery.” [TBA #2, practicing for over 60 years]

“Most of the men do not have any good relationship with their wives. They don't care whether they go to the hospital or to the TBA.” [TBA #4, practicing for 15 years]

“Some do but majority do not support their spouses. They do not care at all.” [TBA #5, practicing for 30 years]

Of the 20 nursing mothers who participated in the study, only three reported receiving support with housekeeping chores and finances from their partners. It should be noted that these were the same participants that reported more than secondary school education:

“When I was four months, I started bleeding when I was sweeping. I went to the hospital and they advised me not to engage in any strenuous activities. So, my children and husband, they perform the house chores. They cook, they sweep and almost everything…They did not allow me do anything. I was just resting till it was time for me to deliver.” [Nursing Mother #14]

“I live with him [husband]. He is supportive…he only helps in cooking. But the other things he does not do them.” [Nursing Mother #17]

“Yes, my husband assists me a lot when he is around. He cooks for us. Anytime I complain about any health issues, he goes to the drug store to purchase medicine for us. He washes at times. It only sweeping that he does not do.” [Nursing Mother #6]

None of the nursing mothers in the study reported physical, emotional or sexual abuse. However, many of them were quick to point out that their partners do not support them financially, detailing the various ways they had to support themselves and their children, from engaging in simple trading like selling bananas, fetching water to farming in the village:

“No, he was not supportive. He does not send anything at all. He does not give me anything. Because, he said I should abort and I refused. [Does he beat you?] No, but he left the house.” [Nursing Mother #11]

“I don't live with my partner. We are married. [Does your partner visit you?] No. he does not visit. [Did he come when you delivered?] Yes. The baby is 1 year 8 months old but he came only once. I live with my brother and he is the one taking care of the child.” [Nursing Mother #8]

“I fetch water for people in their various homes. I also sell bananas to support me and my children.” [Nursing Mother #5]

“I sent the first child to one of my aunties so that I can take care of myself. I started selling small, small and that was what I was supporting myself till now.” [Nursing Mother #2]

Mothers with more experience of childbirth were more likely to report lack of financial support during pregnancy and childbirth. Mothers who reported lack of financial support did not view lack of financial support as abuse, rather as hinderance to their efforts to care for their children, saying “*…he's not beating me*.” It should be noted that when participants were asked what they think would happen if they insist on housekeeping assistance, and especially financial support from their partners, a number of them laughed sarcastically, and a participant said, “*why bother*?”

### 4.2 Traditional birth attendants as mediators

Traditional birth attendants' descriptions of the situations nursing mothers face during pregnancy paints a grim picture of the reality of women's lives in their most vulnerable state, especially in rural communities. Many sought out traditional birth attendants' services due to lack of resources to visit the local healthcare facilities, or teenage pregnancy:

“The response over the years has been that they will not be able to be visiting the hospital all the time for their antenatal care so since they are in the village and the community with them, they would want [me] to be helping them.” [TBA #1, practicing for 20 years]

“I don't know why they prefer to come to me but I think unavailability of health facilities during these days was one of the reasons. Also, the teenagers who get pregnant don't know anything about pregnancy and the care for pregnant women so they come to me for advice/counseling.” [TBA #2, practicing for over 60 years]

“I have realized that pregnant adolescent girls don't know what to do because they are young, they don't even know who impregnated them so they come to us for advice. They also don't like going to the hospital because they don't have money so they come to us.” [TBA #4, practicing for 15 years]

Since traditional birth attendants are part of the community, it provides them unfiltered access to observe the interactions between pregnant women and their partners. TBAs provided more candid details about the financial situation of pregnant women, specifically how their mates provide no financial support:

“Some of the women have never been to the hospital. When I ask them why, their response is that their husbands do not give them money that's why they don't go. So, I insist on them coming with them [husbands] so I can talk to the men to take good care of their wives.” [TBA #3, practicing for 10 years]

“Most of the men do not have any good relationship with their wives. They don't care whether they go to the hospital or to the TBA. But when we talk to the women to come with them [men], we advise them [men], and tell them about their roles, we see that things get better.” [TBA #4, practicing for 15 years]

“Some of the women even come without items and cloth. Sometimes I have to give out my cloth and other items which they don't even return to me. [What do you do when they say they cannot pay?] You just let them go because if you insist, it means she will stay with you and you have to feed her and she becomes a burden.” [TBA #6, practicing for 5 years]

“Some do but majority do not support their spouses. They do not care at all. In some of the cases where I need to refer them to the hospital, the husbands complain of not having any money so sometimes I have to give them money for transport fare to the hospital.” [TBA #5, practicing for 30 years]

“The relationship isn't good. The husbands do not support their wives financially, so their wives do not like going to the hospital, but because they do not pay all the time for the services of TBAs, they prefer coming to us.” [TBA #7, practicing for 1 year]

The words of the oldest traditional birth attendant in the study (81 years old), captured the feeling of the group about their observations of pregnant women and their partners, while also exemplifying their role as mediator in the community:

“I don't attend to those who come without their husbands, I only attend to them if they come with their husband. Some do not help their wives so I give advice to those men who do not support their wives. I tell them what they are to do during pregnancy and delivery. I see some changes after talking to them. [TBA 2, practicing for over 60 years]

Participants discussed improvement they observed when they step in as mediators, with the women reporting back to them the change in their partners:

“I just counsel them and I see changes, sometimes the women come back with good reports on their husband's support.” [TBA #1, practicing for 20 years]

“When we talk to the women to come with them [men], we advise them [men], and tell them about their roles, we see that things get better.” [TBA #4, practicing for 15 years]

It is interesting that only one TBA mentioned seeing some men beat their wives, and she stepped in to mediate, and reported that such men stopped: “[Have you ever seen a man beating the wife?] I have seen some do that [beat their wives] but when I call the men and talk to them, they don't do it anymore.” [TBA #5, practicing for 30 years]

## 5 Discussion

This study presented nursing mothers' perceptions of IPV and traditional birth attendants’ role in mediating such events using the social constructivist framework. It should be noted that the rationale for the study was not to examine IPV, but rather to explore how nursing mothers describe support and how they were responding to the ban on traditional birth attendants which a number of them seek care and delivery services from. Ghana banned traditional birth attendants following the WHO policy change that safe motherhood is best achieved when women receive care from skilled attendants, which were classified as people with midwifery skills (nurses, midwives and doctors). However, it became obvious during the interviews and discussions that the challenges nursing mothers experience had more to do with lack of support from their partners, in terms of housekeeping chores, and especially with finance, which seems to be the primary factor in seeking TBA services. Most participants in the study reported lack of support with housekeeping chores from their husbands and partners, along with lack of financial support to care for their needs and that of their children. While pregnancy in itself can be physically and emotionally demanding on a woman's body, adding the responsibility of housekeeping and the uncertainty about financial support exacerbates the stress the woman is under, and can complicate her pregnancy journey (41).

Challenges experienced by participants during their pregnancy were confirmed by traditional birth attendants' observations. Pregnant women present themselves for care and delivery to traditional birth attendants without any money for the services they sought. They sought TBA services without the basic necessities for their baby such as blankets or clothes or even soap. Some husbands' assertions about having no money such that TBAs provide funds to cover transportation cost to the hospital in situations that TBAs feel the women should seek medical care paints a grim picture about pregnant women's situation. It can be argued that the reality they faced with lack of financial support from their husbands/partners meant they had to seek affordable and accessible means to care for themselves during pregnancy—seeking the services of traditional birth attendants. Research have shown that support during pregnancy is very important for successful pregnancy and delivery, and essential for the good health of the mother and child; and decreased the odds of IPV during pregnancy (42–44). At the same time, the lack of support can have devastating effects on mother and child (45).

Cohen and colleagues (46) described the various types of support women need during pregnancy from provision of emotional support (e.g., caring) to instrumental support, such as helping with housekeeping chores, to practical support such as financial support. Gjerdingen and others (47) concluded that emotional and tangible support provided by the spouse is related to the wellbeing of the pregnant women, while Schaffer and Lai-Hoagberg (48) reported that “social support provided by the partner correlated positively with adequacy of prenatal care” in their study on the effects of prenatal care and health behaviors of low-income women. As such, as important as it is for pregnant women to receive support, it is also very important who is providing the support. The role of significant others, like husbands and partners are crucial to the health and wellbeing of the mothers-to-be. While the results from this study cannot speak directly to how critical it is for husbands and partners to provide support to pregnant mothers, the fact that their lack of support presented additional burden on nursing mothers is demonstrated in the findings.

Though some women in the study reported lack of housekeeping and financial support, none reported experiencing emotional, physical or sexual abuse, with some stating: “*he's not beating me*.” The women did not view lack of financial support as abuse, rather as hinderance to their efforts to care for their children. This is similar to findings by Dako-Gyere and colleagues (49) in their research on community perception of IPV in the central region of Ghana. Their findings indicated that female participants linked IPV financial abuse to the children and family economy. It should also be noted that participants' perception of IPV as physical abuse is in line with most descriptions of IPV. Anecdotal accounts from women outside of the study participants by the first and second authors align with the socio-cultural perception that IPV is physical and verbal, but not necessarily sexual. In fact, the idea that people in a marriage or consensual relationship can be subjected to sexual abuse is one that is slowly gaining acceptance in urban areas, but not so much in rural communities. In Ghana, marital rape is implied in the 2007 Domestic Violence Act, but not explicitly stated as a crime (49). Attributing the perception of non-consensual sexual acts in a marriage to patriarchy, payment of bride price, privatization of marital sexual abuse among many others, Adodo-Samani (50) reported that only 3% of males and 18% of females in her study perceive non-consensual sexual acts in marriage as rape, and concluded that while the “perception of marital rape [is] factual, [it] does not necessarily translate into the acceptability of the criminalization of the phenomenon.”

Women demand for financial support, especially to care for their children can escalate into physical abuse. This may explain why women in the study laughed sarcastically when asked what they think would happen if they insist on financial support from their partners, and they responded with “*why bother?*” It is also of note that though one of the TBA participant mentioned observing pregnant women being physically abused, there is no way to confirm if it was one or more of the nursing mothers in the study. Unfortunately, most research on IPV focus on the physical, emotional or sexual abuse, with less focus on the financial aspect, or what Postmus and colleagues (51) described as the “invisible form of intimate partner violence.” A review of IPV descriptions by various national and global organizations and institutions focused on the physical, emotional and sexual abuse of IPV, with no mention of financial abuse as a form of IPV (52). In fact, in their multi-country review of economic abuse as a form of IPV, Postmus and colleagues (51) reported that of all the forty-six peer-reviewed studies they found, only twenty articles “included a clear definition of economic/financial abuse and/or listed more than one tactic used by perpetrators to illustrate the construct in the introduction or background section of the manuscript.” Only eight studies in the 46 studies came from Sub-Saharan Africa, and none of the eight studies had a clear definition of economic and/or financial abuse, or included tactics nor capture the construct. It was concluded that a lack of consistency about the definition of economic and/or financial abuse, and agreed upon index on how to measure it has hindered research despite how widespread the issue is. If financial abuse is not part of the main discourse in the description of IPV, then it presents a challenge in capturing how rampant it is in serving as a precursor to physical, emotional and sexual abuse in sub-Saharan, and can undermine efforts to intervene in protecting women, especially pregnant and nursing mothers. While there is still dearth of research on how financial abuse can be defined and/or measured (51), it is well documented in research that it is one of the tactics used by an abuser to maintain control (53).

Traditional birth attendants in the study described situations in which they had to mediate between pregnant women and their husbands and partners. Keeping in mind that most pregnant women and teen girls sought out their services due to lack of funds to pay at the hospitals (33), TBAs find themselves performing more services that goes beyond care and delivery—the role of a mediator and counselor. Gbogbo and others (53) reported that TBAs serve as mother figures to teenage mothers providing emotional and financial support. Ana (55) argued that TBAs offer more than care and delivery because they are part of the community, “they understand the traditions, cultures, and languages of the women that they attend to,” and are well respected and trusted members (26, 32, 33). As such, they have unfiltered access to what pregnant women are going through because they are on the ground, besides the fact that they themselves may be mothers. In this study, traditional birth attendants' readiness and willingness to counsel husbands, to mediate disagreements, and even provide hospital transportation funds to support pregnant women in dire need of biomedical intervention speaks to the position they hold in the community. Adatara and colleagues (34) findings that TBAs arrange for transportation and accompany women in labor to health facilities are similar to findings in this study.

Furthermore, results from this study confirm the various tangible and intangible ways traditional birth attendants are assets in their communities similar to findings on the role of TBAs in care and delivery in limited resource settings (26, 32–34, 55). While one of the aims of this study was to explore how TBAs in rural communities of Hohoe are coping since the ban of their services, it became quite obvious that their contribution is not always limited to providing care and delivery. The fact that they can mediate between spouses about financial support, and even follow-up with the women about how things are between them and their mates speaks to how involved they are in the community, and how they see their roles. This is in addition to the services identified by Adatara and others (34) that TBAs provide, which include counseling to women during pregnancy and childbirth, and education to couples on natural family planning methods. While not presented in this paper, all the TBAs in the study described receiving food items as form of payment for their services, though they themselves don't have much in the first place, which is in line with earlier research (33, 34). Some TBAs in the study discussed not insisting on payment or letting new mothers stay with them until they [new mothers] can pay because “*it means she will stay with you and you have to feed her and she becomes a burden*.” The words of the oldest TBA (82 years old) in the study best capture the sentiments of all TBA participants: “*I cannot turn them away if they come to me. I do it for God.*” Unfortunately, the focus on unsanitary situations in which some TBAs provide care and delivery have overshadowed other ways in which they are invaluable to the communities they serve (55). It can be argued that a participant comment that she will not attend to a pregnant woman without her husband accompanying her exemplifies TBAs' recognition of how important their role is to pregnant women, to their communities and to protecting women like themselves.

The findings from this study are context specific to the women that participated in the discussions from the rural communities in Hohoe, specifically Atabu, Godenu, Kledzo, Abenyinase, Wegbe, and Bla communities, and cannot be generalized to women in other settings. Also, the findings are based on self-reports from a small group of participants, and there is no way to confirm their comments about not experiencing physical violence from their mates. However, there are shared commonalities in the perceptions of nursing mothers about intimate partner violence and the observations of TBAs and their roles in rural communities based on findings from published research in Ghana specifically, and in other parts of Sub-Saharan Africa, which findings from this study augment. Their perceptions of intimate partner violence provide a useful context in exploring how socio-cultural construction can influence meanings attached to experiences and create a disconnect between how the problem is defined vs. how is it experienced. In addition, this study addressed a gap in the role of traditional birth attendants as *mediators* in the context of intimate partner violence and maternal and child health from the perspectives of mothers. More research is needed to examine fathers' perspectives of TBAs.

Maternal and Child Health is a particularly sensitive measure of a nation's health as it lays bare the indicator for the success of the nation and its future. Lu (56) described maternal and child health as “what we do together as a society to ensure the conditions in which women, children, and families can be healthy.” While scientific and medical research has improved care, delivery, and support for women and children, the impact of IPV have certainly undermine such efforts. There is no doubt that there are grave concerns about improving birth outcomes for mothers and children, and that lack of adequate and proper support will further undercut the attainment of one of the primary goals of 2,030 sustainable developments: gender equality and empowerment of women and girls (1, 57). Therefore, interventions to address intimate partner violence should take into account the socio-cultural construction of such experiences by women. Language of intervention is very salient in IPV prevention/intervention endeavors as a disconnect between the way IPV is defined and the way it is perceived by “victims” may weaken such activities. In addition, traditional birth attendants must be considered part of the safety net fabric in improving maternal and child health in rural communities as pregnant women and nursing mothers depend on them for survival, no matter how unskilled they may be. Until every rural community can have a skilled attendant (nurses, midwives and doctors), there is a need to provide training for traditional birth attendants in order to improve maternal and child health.

## Data Availability

The raw data supporting the conclusions of this article will be made available by the authors, without undue reservation.
